# Chronic low-grade inflammation in patients with systemic sclerosis is associated with increased risk for arteriosclerotic cardiovascular disease

**DOI:** 10.3389/fmed.2024.1446268

**Published:** 2024-11-05

**Authors:** Ursula Heilmeier, Daria Feldmann, Andrew Leynes, Magdalena Seng, Ilona Jandova, Marius Keute, Florian Kollert, Reinhard Edmund Voll, Stephanie Finzel

**Affiliations:** ^1^Department of Rheumatology and Clinical Immunology, Faculty of Medicine, Medical Center–University of Freiburg, University of Freiburg, Freiburg im Breisgau, Germany; ^2^Musculoskeletal Quantitative Imaging Research Group, University of California San Francisco, San Francisco, CA, United States; ^3^Department of Anesthesiology, Krankenhaus Porz am Rhein, Cologne, Germany; ^4^Department of Radiology and Nuclear Medicine, University of Basel, Basel, Switzerland; ^5^Institute for Neuromodulation and Neurotechnology, University of Tübingen, Tübingen, Germany; ^6^Department of Rheumatology and Immunology, University Hospital Bern, University of Bern, Bern, Switzerland; ^7^Department of Rheumatology, University Hospital Basel, Basel, Switzerland

**Keywords:** systemic sclerosis, CRP, arteriosclerotic cardiovascular risk, carotid ultorasonography, inflammatory systemic sclerosis, Framingham score, ASCVD risk score, SCORE (systemic coronary risk evaluation)

## Abstract

**Background:**

Vasculopathy is a hallmark of systemic sclerosis (SSc) putting patients at an increased risk of cardiovascular disease. Approximately 20–25% of all SSc patients show prolonged elevated C-reactive protein (CRP) levels and thus signs of chronic low-grade inflammation. While CRP−positivity is an independent predictor of cardiovascular disease in non-SSc populations, the relationship between CRP−positivity and cardiovascular health/atherosclerosis in SSc patients is only incompletely understood. Here, we aimed to assess (1) which general, SSc disease-specific and cardiovascular parameters are associated with CRP−positivity in a cohort of SSc patients with prolonged CRP elevations (CRP+ SSc group) relative to SSc patients without CRP elevations (CRP− SSc group). In addition (2), we aimed to investigate whether prolonged CRP−positivity in SSc patients is associated with a higher cardiovascular risk and an increased atherosclerotic burden. We also aimed to (3) identify via random forest classification modeling which combined cardiovascular and/or SSc-specific parameters could differentiate best between SSc patients with elevated CRP levels (the so-called “inflammatory SSc subtype”) and SSc patients without increased CRP levels.

**Methods:**

Sixty-five SSc patients were recruited and assigned to the CRP+ SSc group (*n* = 20) if their CRP levels were > 5 mg/L in at least three half-yearly visits within 2 years before enrolment or to the CRP− SSc group (*n* = 45), respectively. All patients underwent an anamnesis, physical examination, blood draw, and bilateral carotid ultrasound in order to assess arteriosclerotic burden including the presence, number and height of plaques, and carotid intima–media thickness (CIMT) as well as lipid profiles. 10-year ASCVD risk was estimated via the ASCVD risk estimator plus. Statistical evaluation included Spearman’s correlations, logistic regression and random forest modeling under 5-fold cross-validation, and permutation testing to determine combinations of cardiovascular variables highly discriminatory for CRP−positivity.

**Results:**

SSc groups showed comparable mean age, height, and extent of SSc organ involvement. Regarding cardiovascular health, CRP+ SSc patients exhibited a significantly altered HDL-, LDL-, and triglyceride profile (0.001 ≤ *p* ≤ 0.017) and a significantly higher 10-year ASCVD risk (*p* = 0.047), relative to CRP− SSc patients. Additionally, within the subgroup of CRP+ SSc patients, positive correlations between CRP levels and CIMT right (*ρ* = 0.657, *p* = 0.002) and mean CIMT left and right (ρ = 0.497, *p* = 0.026) were seen. Combined ROC models identified the four lipid components (HDL, LDL, total cholesterol, and triglycerides) or the SSc duration and ASCVD category to differentiate with high cross-validated ROC-AUCs (AUC: 0.83 ± 0.15, and AUC: 0.86 ± 0.09, *p <* 0.001) for prolonged CRP−positivity among SSc patients.

**Conclusion:**

Our data indicate that persistent CRP−positivity and thus chronic low-grade inflammation in SSc patients enhance the risk for arteriosclerotic-cardiovascular disease significantly beyond the ASCVD risk observed for our SSc patients without CRP elevations. It seems to be along with a disrupted lipid profile the hallmark of a distinct “inflammatory” subgroup of SSc patients. However, large population-based studies and clinical trials in patients with SSc are needed to validate our findings in a prospective or interventional setting.

## 1 Introduction

Systemic sclerosis (SSc) is an autoimmune disease characterized by excessive deposition of extracellular matrix resulting in distinct progressive fibrosis of the skin and of internal organs, such as the lungs, and in vasculopathy (1). SSc is linked to one of the highest mortality rates among all rheumatic diseases (2). Clinical diagnosis, treatment, and research are challenging due to the phenotypic heterogeneity of SSc. Efforts have been undertaken to identify biomarkers that can help predict SSc organ involvement and severity, SSc progression, and mortality and may therefore be utilized for risk stratification and personalized treatment.

C-reactive protein (CRP) is an easily accessible and cost-efficient serum marker of inflammation in daily clinical routine. To date, several studies have shown that approximately 23.2–41.5% of patients with SSc have elevated CRP levels (3, 4). In approximately 18% of SSc patients, CRP elevations are even persistent (5, 6). More importantly, prospective studies and a meta-analysis carried out in this particular “inflammatory subtype” of SSc patients have linked the presence of elevated CRP levels (cutoff >5 mg/L) to worse health outcomes such as an increased risk of pulmonary hypertension (7), progressive fibrosis, and higher mortality (8, 9). In addition, elevated CRP levels have been found associated with the increasing severity of skin and joint involvement (9, 10).

Microvascular and macrovascular diseases are additional hallmarks of SSc, putting SSc patients at a significantly increased risk of cardiovascular events compared to the general population (11, 12). In a recent meta-analysis, pooled hazard ratios in SSc patients ranged from 2.36 (95% CI 1.97–2.81) for cardiovascular disease (CVD) or myocardial infarction (95% CI 1.71–3.25) to a hazard ratio of HR 5.27 (95% CI 4.27–6.51) for peripheral vascular disease compared to the general population (12). For most inflammatory rheumatic diseases, an increased cardiovascular risk has been demonstrated with the most detailed data available for rheumatoid arthritis (RA). Analyzing carotid intima–media thickness (CIMT) and pulse wave velocity, Dimitroulas et al. (13) found comparable magnitudes of subclinical atherosclerosis in patients with SSc when compared with patients with RA, highlighting the importance and need for cardiovascular risk assessment and management also for SSc patients (13). While CRP elevation has long been established as a strong predictor and risk factor for carotid arteriosclerosis (14), future cardiovascular events (15), and poor prognosis after CV events (16, 17) in non-SSc patients, the relationship between CRP levels and cardiovascular health/arteriosclerosis in SSc patients has to date only been incompletely (18) and inconsistently (19–23) investigated as studies so far either assessed CRP–cardiovascular correlations only as a (bystander) secondary outcome (19) or their study design and sample size did not allow for or foresee more in-depth analyses (such as stratification by prolonged CRP status) (13, 22–25).

Therefore, the aims of this cross-sectional observational study were 3-fold: (1) to determine in a cohort of SSc patients with prolonged CRP elevations (CRP+ SSc group) relative to SSc patients without CRP elevations (CRP− SSc group) which general, and/or SSc disease-specific, and/or cardiovascular parameters are associated with a prolonged CRP elevation in systemic sclerosis. Furthermore, we aimed (2) to investigate whether prolonged CRP−positivity in SSc patients is associated with a higher cardiovascular risk as determined via the Framingham risk and arteriosclerotic cardiovascular disease (ASCVD) risk scores and with an increased atherosclerotic burden as determined by carotid ultrasound. We also aimed to (3) identify via random forest classification modeling, which combined cardiovascular and/or SSc-specific parameters could differentiate best between SSc patients with elevated CRP levels (the “inflammatory SSc subtype”) and SSc patients without increased CRP levels.

## 2 Materials and methods

### 2.1 Patient selection

In this cross-sectional, observational study, 65 patients with SSc from the outpatient clinic at the Department of Rheumatology and Clinical Immunology, University Medical Center, Freiburg, Germany, were enrolled. All patients fulfilled the ACR/EULAR 2013 classification criteria for SSc at the time of enrollment (26). Inclusion and exclusion criteria for this study as well as the group assignment into CRP−positive and CRP−negative groups have been published elsewhere (10). As described (10), 20 of the 65 patients exhibited elevated CRP levels >5 mg/L in at least three half-yearly visits within 2 years prior to enrolment and were assigned to the CRP−positive group (CRP+ SSc group), while 45 SSc patients exhibited normal CRP concentrations for at least 1.5 consecutive years before enrollment and were assigned to the CRP−negative group (CRP− SSc group). The Freiburg Institutional Review Board granted ethical approval for the study (386/17). Prior to any study-related measures, all patients provided written informed consent. The study was carried out in accordance with the principles of Good Clinical Practice (GCP) as developed by the International Conference of Harmonization (ICH) and set forth by the Declaration of Helsinki.

### 2.2 Study visits and clinical measurements

At the time of the study visit, all patients underwent a structured interview that assessed traditional risk factors for arteriosclerosis (such as the history of smoking, number of pack years, presence of arterial hypertension, type 2 diabetes mellitus, chronic kidney disease, and a positive family history with respect to the cardiovascular event [any cardiovascular event before the age of 65 years]). Information on the duration and extent of SSc and current medication use was extracted from the patient charts. In addition, all patients underwent a physical examination. This included apart from height, weight, heart rate, and blood pressure, the assessment of the acra for the presence of digital ulcerations, Raynaud’s syndrome, and a tender and swollen joint count, which were recorded in a standardized way.

### 2.3 Laboratory measurements

All patients underwent a blood draw at the time of the study visit, and serum antibody and CRP concentrations were measured at the Department of Rheumatology and Clinical Immunology University Medical Center Freiburg laboratory (10). In addition, data on fasting serum levels of total cholesterol, HDL cholesterol, LDL cholesterol, total triglycerides, and eGFR levels were retrieved from the patients’ laboratory charts, which all had been measured by the University Medical Center Freiburg’s Department of Clinical Chemistry and Laboratory Medicine laboratory according to the quality standards of routine clinical diagnostic laboratory measures.

### 2.4 Ultrasound evaluation of carotid arteries

At the study visit, bilateral carotid ultrasound was carried out at the common and external carotid arteries using an 18-MHz linear array transducer on the same Esaote MyLab Twice ultrasound scanner (Esaote, Genoa, Italy) by the same sonographer (SF) with 9-year experience in ultrasound at the time of examination. Carotid intima–media thickness (CIMT) was measured in millimeters as a well-established marker of arteriosclerosis and determinant of cardiovascular disease (27, 28). CIMT measurements were performed by the American Society of Echocardiography’s consensus scanning protocol (29). Common and external carotid arteries were scanned and evaluated in both the longitudinal and axial plane for the presence, height, and number of arteriosclerotic plaques at standardized locations as suggested by the American Society for Echocardiography. An arteriosclerotic plaque was therefore defined as a focal thickening of the arterial wall that was at least 50% thicker than the surrounding vessel walls and/or a focal thickening of the arterial wall protruding into the vessel lumen and being bigger than 1.5 mm (29). In order to not overlook cholesterol plaques, which are difficult to visualize in B-mode, color Doppler ultrasound scanning at 5.6 MHz was utilized. In total, the following arteriosclerotic ultrasound parameters were acquired for the left and right carotid arteries separately: carotid intima–media thickness (CIMT) in millimeters of the left and right common carotid arteries, the maximum plaque height in millimeters, and the number of plaques (n). In addition, the total number of plaques was calculated as a summary variable summarizing the number of all plaques of both sides. A mean CIMT of left and right carotid arteries was calculated as the sum of left and right CIMT divided by two and reported in millimeters.

### 2.5 Estimation of arteriosclerotic cardiovascular risk via Framingham, ASCVD, and SCORE 2019 risk scores

In order to estimate the arteriosclerotic cardiovascular risk in both SSc groups, three different risk calculators were utilized. For the individual 10-year risk of hard coronary heart disease (individual FRS 10-year risk of HCDH) and the average 10-year risk of hard coronary heart disease (average FRS 10-year risk of HCDH), we used the Framingham Risk Score for Hard Coronary Heart Disease risk calculator/outcome model, which is freely available online under the URLs https://www.mdcalc.com/calc/38/framingham-risk-score-hard-coronary-heart-disease and the https://www.framinghamheartstudy.org/fhs-risk-functions/hard-coronary-heart-disease-10-year-risk/ which is based on risk equations published by Wilson et al. (30). Outcome is the 10-year risk of hard coronary heart disease (=defined as any myocardial infarction or coronary death). The model is intended for use in patients aged 30–79 years with no prior history of coronary heart disease or intermittent claudication.

To estimate a patient’s 10-year risk for atherosclerotic cardiovascular disease (ASCVD), we utilized the ASCVD Risk Estimator Plus provided by the American Heart Association and the American College of Cardiology and freely accessible online under the URL https://tools.acc.org/ascvd-risk-estimator-plus/#!/calculate/estimate/. Atherosclerotic cardiovascular disease (ASCVD) is hereby defined as coronary death or non-fatal myocardial infarction, or fatal or non-fatal stroke, and the calculator is based on the pooled cohort equations and on the study published by Goff et al. (31) and others (32, 33). The input information needed to estimate ASCVD risk includes age, sex, race, total cholesterol, HDL cholesterol, systolic blood pressure, blood pressure-lowering medication use, diabetes status, and smoking status. Depending on their calculated ASCVD 10-year risk, all SSc patients were stratified in line with the recommendations of the American College of Cardiology into one of the following four ASCVD risk groups: low ASCVD risk group (containing SSc patients with <5% calculated ASCVD 10-year risk score), borderline ASCVD risk group (containing SSc patients with a 5–7.4% calculated ASCVD 10-year risk score), intermediate ASCVD risk group (containing SSc patients with a 7.5–19.9% calculated ASCVD 10-year risk score), and high ASCVD risk group (containing SSc patients with a ≥ 20% calculated ASCVD 10-year risk score).

To estimate a patient’s 10-year risk of fatal cardiovascular disease (CVD), we utilized the Systematic Coronary Risk Evaluation (SCORE) cardiovascular risk chart (SCORE 2019) as provided and developed by the European Society of Cardiology and as published and updated by Mach et al. (34). We employed the low-risk chart as Germany is considered a low-risk country (34). For the SCORE 2019 risk calculation, the input information encompassed the following risk factors: age, gender, smoking, systolic blood pressure, and total cholesterol levels.

### 2.6 Statistical evaluation

The normal distribution of data was visually verified via Q-Q plots and statistically tested using Kolmogorov–Smirnov and Shapiro–Wilk testing. For normally distributed data, differences between CRP+ and CRP− groups were assessed via independent *t*-tests, Pearson’s chi-squared tests, or Fisher’s exact tests, as appropriate. For non-normally distributed data, Mann–Whitney *U*-tests were utilized. Spearman’s rank order correlations were performed in order to assess the associations between CRP levels and cardiovascular risk factors, risk scores, and cardiovascular carotid ultrasound parameters in the overall cohort and the CRP+ and CRP− groups. Due to the relatively small patient sample, we could not test all variables in a common statistical model. Instead, we used binary logistic regression models to test the associations between the dependent variable (CRP positive yes/no) and the general, cardiovascular, and arteriosclerotic carotid ultrasound parameters. An odds ratio (OR) >1 indicates that a higher value in the respective candidate predictor variable (or response “yes” for categorical candidate predictor variables) is associated with a higher probability of belonging to the group CRP+. Subsequently, we used Random Forest classification along with a greedy feature selection procedure (35) with a 5-fold cross-validation and permutation test with 1,000 permutations in order to determine the optimal combination of cardiovascular variables associated with a positive CRP status. The binary classification area under the receiver operating curve (ROC-AUC) was employed as the scoring metric for feature selection. K-nearest neighbor imputation was performed ahead of any missing values. The AUCs between combined ROC models across the 5-fold cross-validation were compared using the Wilcoxon signed-rank test. Data analysis was carried out in Python using the scikit-learn 1.3.0 package and using SPSS statistics IBM, version 25. Statistical significance was defined as *p <* 0.05.

## 3 Results

### 3.1 Patients’ characteristics

Patient demographics, SSc disease characteristics, and information on SSc-related medication usage as well as laboratory measures are summarized in Tables 1A,B and have in part been previously published by Feldmann et al. (10). In brief, CRP+ and CRP− groups showed similar mean age and height and did not significantly differ with respect to their age of SSc onset and gender proportions and with respect to the extent of their SSc organ involvement. Particularly, the proportions of SSc patients suffering from SSc-related vascular complications such as digital ulcerations, Raynaud’s syndrome, or pulmonary arterial hypertension were comparable among both CRP− groups, as were the proportions of vasodilatory drug users (*p* > 0.05). However, CRP+ SSc patients had a significantly higher BMI (28.2 vs. 25.8 kg/m^2^, *p* = 0.037), used significantly more often glucocorticoids (*p* = 0.023), significantly less often hydroxychloroquine (*p* = 0.012) and showed a statistical trend toward having on average an almost 2-year shorter SSc disease duration. As outlined previously (10), CRP+ SSc patients had a higher frequency of being SCL70-antibody positive (*p* = 0.041), whereas the CRP− SSc patients showed significantly more often positive anti-nucleosome antibodies (*p* = 0.013).

**Table 1:** Patient characteristics of all 65 study participants with systemic sclerosis (SSc), presented by CRP status.

(A)			
	Means ± SD		
	CRP+ group (*n* = 20)	CRP− group (*n* = 45)	CRP+ vs. CRP− *p*-value
Demographics			
Age [years]*	59.3 ± 13.0	58.0 ± 13.9	0.984
BMI [kg/m^2^]	28.2 ± 5.3	25.8 ± 4.9	**0.037**
Height (m)	1.7 ± 0.1	1.7 ± 0.10	0.772
Weight [kg]	79.6 ± 19.1	71.7 ± 15.5	*0.085*
Sex, female *n* [%]*	13 [65.0]	38 [84.4%]	0.105
*SSc characteristics*			
Time since SSc diagnosis [months]*	107.2 ± 114.6	130.9 ± 91.6	*0.093*
Age of SSc diagnosis [years]*	50.4 ± 12.3	47.1 ± 14.43	0.453
Type of SSc Limited *n* [%]*	9 [45.0]	28 [62.2]	0.278
Type of SSc Diffuse *n* [%]*	11 [55.0]	17 [37.8]	0.278
Overall SSc organ involvement ł *n* [%]*	15 [75.0]	27 [60.0]	0.276
Heart involvement *n* [%]*	1 [5.0]	1 [2.2]	0.524
Lung involvement *n* [%]*	10 [50.0]	16 [35.6]	0.289
Presence of PAH *n* [%]	6 [30.0]	10 [22.2]	0.542
Presence of SSc-related interstitial lung disease [%]	7 [35.0]	9 [20.0]	0.223
Esophagus involvement *n* [%]*	10 [50.0]	19 [42.2]	0.598
Gastrointestinal involvement *n* [%]*	1 [5.0]	3 [6.7]	1.000
Kidney involvement *n* [%]*	0 [0.0]	2 [2.2]	1.000
Presence of digital ulcerations *n* [%]	5 [25.0]	8 [17.8]	0.517
History of digital ulcerations *n* [%]	6 [30.0]	14 [31.1]	0.547
Presence of Raynaud’s syndrome	20 [100.0]	42 [93.3]	1.000
Medication usage			
Usage of immunosuppressants			
glucocorticoids *n* [%]*	8 [40.0]	6 [13.3]	**0.023**
hydroxychloroquine *n* [%]*	0 [0.0]	12 [26.7]	**0.012**
methotrexate *n* [%]*	2 [10.0]	2 [4.4]	0.581
mycophenolate mofetil *n* [%]*	3 [15.0]	8 [17.8]	1.000
azathioprine *n* [%]*	1 [5.0]	1 [2.2]	0.524
cyclophosphamide *n* [%]*	1 [5.0]	0 [0.0]	0.308
Usage of vasodilatory medication	14 [70.0]	29 [64.4]	0.379
endothelin receptor antagonists *n* [%]	4 [20.0]	5 [11.1]	0.262
PDE-5 inhibitors *n* [%]	2 [10.0]	2 [4.4]	0.571
calcium antagonists *n* [%]	12 [60.0]	25 [55.6]	0.573
Antihypertensive medication usage ~	9 [45.0]	21 [46.7]	0.515
Lipid-lowering agents^ǂ^	0 [0.0]	6 [13.3]	0.120
Ł organ involvement was considered present if any of the following organs (esophagus, lung, heart, GI system, or kidney) were affected by SSc. *Reprinted with permission as in Feldmann et al. (10) PAH, pulmonary arterial hypertension. ~ includes antihypertensive classes beta blockers, thiazide diuretics, loop diuretics, sartans, ACE-inhibitors. ^ǂ^Includes statins, fibrates, and ezetimibe. Shown are (unadjusted) means with standard deviations (SD). Significant p-values (p < 0.05) are marked in bold print, and statistical trends are printed in italics. CRP status was considered positive or negative, respectively, if at least 75% of the highly sensitive CRP values were positive (> 5 mg/L) or negative (≤ 5 mg/L) in at least three half-yearly visits within the past 2 years prior to the start of the study.			

(B)	Means ± SD		
	CRP+ group (*n* = 20)	CRP− group (*n* = 45)	CRP+ vs. CRP− *p*-value
Serum laboratory measures			
CRP levels [mg/l]	11.7 ± 8.0	3.5 ± 0.5	**<0.001**
Anti-Scl-70 antibody positive *n* [%]*	10 [50.0]	10 [22.2]	0.041
Anti-centromere antibody positive *n* [%] *	7 [35.0]	31 [68.9]	0.013
eGFR (estimated glomerular filtration rate) [ml/min/1.73m^2^]	80.5 [61.1–87.1]	77.0 [73.6–85.3]	0.439
Total cholesterol [mg/dl]	216.7 ± 34.0	206.4 ± 41.0	0.330
HDL cholesterol [mg/dl]	50.7 ± 16.8	67.4 ± 16.4	**<0.001**
LDL cholesterol [mg/dl]	152.9 ± 28.4	129.3 ± 37.9	**0.017**
Triglycerides [mg/dl]	186.1 ± 91.1	123.4 ± 54.1	**0.003**
Cardiovascular risk factors			
Arterial hypertension *n* [%]	10 [50.0]	21 [46.7]	0.804
History of smoking *n* [%]	11 [55.0]	18 [40.0]	0.262
Pack years [years]	9.3 ± 15.0	7.5 ± 13.8	0.365
Diabetes mellitus type 2 *n* [%]	4 [20.0]	2 [4.4]	**0.049**
Chronic kidney disease *n* [%]	4 [20.0]	6 [13.3]	0.492
Positive family history of cardiovascular event below the age of 65 years *n* [%]	5 [25.0]	9 [20.0]	0.651
Individual FRS 10-year risk of HCHD [%]	9.5 ± 8.6	6.4 ± 6.9	0.200
Average FRS 10-year risk of HCHD [%]	7.6 ± 5.2	7.8 ± 6.0	0.762
ASCVD 10-year risk [%]	15.0 ± 12.0	9.3 ± 10.3	**0.047**
SCORE 2019 10-year risk of fatal CVD [%]	3.3 ± 3.4	1.9 ± 2.7	**0.040**
Arteriosclerotic assessment by carotid US			
Right-side carotid arteries	(n= 20)	(n=44)	
CIMT right [mm]	0.65 ± 0.19	0.60 ± 0.20	0.212
proportion of patients with right plaques *n* [%]	12 [60.0]	18 [40.9]	0.156
number of plaques right *n*	0.70 ± 0.66	0.48 ± 0.63	0.171
maximal plaque diameter right [mm]	1.35 ± 1.40	0.83 ± 1.11	0.138
Left-side carotid arteries	(*n* = 19)	(*n* =44)	
CIMT left [mm]	0.68 ± 0.29	0.63 ± 0.26	0.349
proportion of patients with left plaques *n* [%]	11 [57.9]	17 [38.6]	0.312
number of plaques left *n*	0.58 ± 0.51	0.43 ± 0.59	0.232
maximal plaque diameter left [mm]	1.22 ± 1.25	1.00 ± 1.55	0.270
Total number of plaques left and right *n*	1.92 ± 0.64	1.67 ± 0.76	0.240
Mean CIMT left and right carotids art. [mm]	0.67 ± 0.19	0.61 ± 0.21	0.313

FRS, Framingham risk score; HCDH, Hard coronary heart disease defined as either myocardial infarction or coronary death; ASCVD, Arteriosclerotic cardiovascular disease; CIMT, Carotid intima–media thickness measured via ultrasound; CVD, cardiovascular disease; SCORE, Systematic cardiovascular risk evaluation. *Reprinted with permission as in Feldmann et al. (10). eGFR is expressed as median [25th–75th percentile].

With respect to cardiovascular health, CRP+ SSc patients showed a significantly altered HDL-, LDL, and triglyceride profile (Table 1B: 0.001 ≤ *p* ≤ 0.017) as well as a significantly higher proportion of type 2 diabetes compared to their CRP− SSc peers (*p* = 0.049). In addition, the estimation of cardiovascular risk scores revealed that CRP+ SSc patients had a significantly higher 10-year risk for ASCVD (*p* = 0.047) than the CRP− SSc patients. Risk stratification of SSc patients by ASCVD risk into a low-ASCVD SSc risk group (<5% ASCVD 10-year risk) and into a combined intermediate-to-high risk ASCVD risk group (IMH risk group, ≥7.5% or > 20% 10y-ASCVD risk estimated) confirmed significantly higher CRP levels in the IMH-risk SSc group compared to low-risk SSc individuals (*p* = 0.024) (Supplementary Table 1). Calculation and intergroup comparisons of SCORE 2019 risk scores demonstrated that the calculated 10-year risk for fatal cardiovascular disease was significantly higher in the CRP+ SSc group relative to the CRP− SSc group (*p* = 0.040). The individual Framingham risk scores for hard coronary heart disease were numerically higher in the CRP+ SSc group but did not reach statistical significance. Average Framingham risk scores for hard coronary heart disease and other cardiovascular risk factors such as the presence of arterial hypertension, chronic kidney disease, smoking history, and positive family history for a cardiovascular event below the age of 65 years were comparable between both groups, as were the atherosclerotic quantitative ultrasound measures of left and right carotid arteries and estimated glomerular filtration rates.

### 3.2 Associations between SSc-specific and demographic parameters, cardiovascular parameters, and CRP status

When assessing the associations between SSc disease-specific, demographic, and cardiovascular parameters with CRP positivity, we found that suffering from type 2 diabetes mellitus increased the risk of having a prolonged CRP−positive status by 5.3-fold (OR: 5.25 [0.87–31.52] *p* = 0.070) (Table 2). Moreover, a 10 mg/dL increase in HDL levels was associated with a 47% reduced risk of having a prolonged positive CRP status (OR: 0.53 [0.36, 0.79] *p* = 0.002) and a 10 mg/dL increase in LDL and triglyceride levels showed a significant 22% or 13% risk increase for being CRP−positive, respectively (*p* = 0.022, and *p* = 0.005). Moreover, a point increase in the 10-year ASCVD risk score resulted in a statistical trend for a 5% risk increase in experiencing sustained CRP−positivity. We also observed statistical trends for weight (OR: 1.03 [1.00–1.06] *p* = 0.089) and BMI (OR: 1.10 [0.99–1.22] *p* = 0.088) to be predictors for CRP−positivity, while other cardiovascular risk factors such as the presence of arterial hypertension or smoking, FRS risk scores and arteriosclerotic ultrasound parameters such as carotid intima–media thickness were not predictive of CRP−positive status (see Supplementary Table 2).

**Table 2:** Results of binary logistic regression analyses were carried out in all *n* = 65 SSc patients to assess associations between SSc disease-specific and demographic parameters, cardiovascular parameters, and CRP−positivity status.

	OR	95% CI of OR	*p*-value
SSc characteristics and demographics			
Time since SSc diagnosis [months]	1.00	[0.99; 1.00]	0.373
Age of SSc diagnosis [years]	1.02	[0.98; 1.06]	0.378
Age [years]	1.01	[0.97, 1.05]	0.729
Height (m)	2.35	[0.01, 680.0]	0.768
Weight [kg]	1.03	[1.00, 1.06]	*0.089*
BMI [kg/m^2^]	1.10	[0.99, 1.22]	*0.088*
Sex (female)	2.92	[0.86; 9.92]	*0.085*
Cardiovascular risk factors			
Total cholesterol [mg/dl]	1.01	[0.99; 1.02]	0.326
HDL cholesterol [10 mg/dL]	0.53	[0.36; 0.79]	**0.002**
LDL cholesterol [10 mg/dL]	1.22	[1.03; 1.44]	**0.022**
Triglycerides [10 mg/dL]	1.13	[1.04; 1.24]	**0.005**
Arterial hypertension *n* [%]	1.14	[0.40, 3.31]	0.804
History of smoking *n* [%]	1.83	[0.64, 5.43]	0.264
Pack years [years]	1.01	[0.97, 1.05]	0.626
Diabetes mellitus type 2 *n* [%]	5.25	[0.87, 31.52]	*0.070*
Chronic kidney disease *n* [%]	1.62	[0.37, 6.49]	0.494
Positive family history of cardiovascular event below the age of 65 years *n* [%]	1.33	[0.36, 4.56]	0.651
Individual FRS 10-year risk of HCHD~ [%]	1.06	[0.98, 1.14]	0.178
Average FRS 10-year risk of HCHD ~ [%]	1.00	[0.89, 1.11]	0.926
ASCVD 10-year risk [%]	1.05	[1.00, 1.10]	*0.078*
SCORE 2019 10-year risk of fatal CVD [%]	1.54	[0.94, 1.47]	0.154

ASCVD, Arteriosclerotic cardiovascular disease; CVD, Cardiovascular disease; FRS, Framingham risk score; ~HCDH, Hard coronary heart disease defined as either myocardial infarction or coronary death; HDL cholesterol, High-density lipoprotein cholesterol; LDL cholesterol, low density lipoprotein cholesterol; SSc, Systemic sclerosis. SCORE, Systematic cardiovascular risk evaluation. CRP status was considered positive or negative respectively, if at least 75% of the highly sensitive CRP values were positive (> 5 mg/L) or negative (≤ 5 mg/L) in at least three half-yearly visits within the past 2 years prior to study start. The odds ratios (OR) along with its 95% confidence intervals are given. Significant *p*-values (*p <* 0.05) are marked in bold print, and statistical trends are printed in italics.

### 3.3 Correlations between baseline CRP levels and cardiovascular risk factors, risk scores, and cardiovascular carotid ultrasound parameters

With respect to clinical and ultrasound correlations (Table 3), we found that in the overall SSc-cohort CRP levels were significant, although weakly, positively correlated with several indicators of cardiovascular health such as HDL cholesterol, LDL cholesterol, triglycerides, the 10-year risk of ASCVD, and the 10-year risks of fatal cardiovascular disease (SCORE 2019) (0.283 < *ρ* < 0.339, *p* ≤ 0.041). In addition, we noted in the overall SSc cohort a weak positive correlation between CRP levels and the right carotid intima–media thickness (CIMT right) (*ρ* = 0.283, *p* = 0.041) and an almost significant correlation between CRP levels and the mean CIMT left and right (ρ = 0.227, *p* = 0.071). When stratifying by the CRP group, both quantitative ultrasound parameters turned into moderate-to-strong positive correlations within the subgroup of CRP+ SSc patients (CIMT right ρ = 0.657, *p* = 0.002, mean CIMT left and right ρ = 0.497, *p* = 0.026). Additionally, moderate positive correlations for ultrasound markers of arteriosclerotic burden, such as the CIMT left and a number of plaques at the right carotid arteries, were found in the CRP+ SSc group, both approaching statistical significance (0.406 < *ρ* < 0.429, *p* ≤ 0.076). In the CRP− group, CRP levels were not correlated with any of the SSc or cardiovascular or ultrasound-specific parameters (data not shown).

**Table 3:** Spearman’s rank-order correlations showing the associations between baseline CRP levels and cardiovascular risk factors, risk scores, and cardiovascular carotid ultrasound parameters in all SSc participants, and for the CRP+ SSc group, respectively.

	All participants (*n* = 65)		CRP+ group (*n* = 20)	
	Correlation coefficient ρ	*P*-value	Correlation coefficient ρ	*P*-value
Age [years]	0.081	0.521	0.399*	*0.081*
Weight [kg]	0.179	0.154	−0.296	0.204
BMI [kg/m^2^]	0.211*	*0.091*	*−0.227*	*0.336*
Total cholesterol [mg/dl]	0.196	0.124	0.175	0.462
HDL cholesterol [mg/dl]	0.293**	0.020	0.288	0.219
LDL cholesterol [mg/dl]	0.326**	0.009	0.172	0.470
Triglycerides [mg/dl]	0.335**	0.008	0.002	0.992
Individual FRS 10-year risk of HCHD [%]	0.118	0.404	−0.011	0.969
Average FRS 10-year risk of HCHD [%]	0.080	0.600	−0.082	0.771
ASCVD 10-year risk [%]	0.274**	0.041	0.086	0.735
SCORE 2019 10-year risk of fatal CVD [%]	0.339**	*0.024*	−0.413	0.112
CIMT right [mm]	0.283**	0.023	0.657**	0.002
number of plaques right *n*	0.185	0.142	0.406*	*0.076*
maximal plaque diameter right [mm]	0.197	0.125	0.236	0.316
CIMT left [mm]	0.114	0.373	0.429*	*0.067*
number of plaques left *n*	0.167	0.192	0.389	0.100
maximal plaque diameter left [mm]	0.151	0.237	0.261	0.281
Total number of plaques left and right *n*	0.140	0.409	−0.310	0.303
Mean CIMT left and right carotids art. [mm]	0.227*	*0.071*	0.497**	0.026

ASCVD, Arteriosclerotic cardiovascular disease; CVD, Cardiovascular disease; CIMT, carotid intima–media thickness measured via ultrasound; FRS, Framingham risk score; HCDH, Hard coronary heart disease defined as either myocardial infarction or coronary death; HDL cholesterol, High-density lipoprotein cholesterol; LDL cholesterol, Low-density lipoprotein cholesterol; SSc, Systemic sclerosis; SCORE, Systematic cardiovascular risk evaluation. Significant *p*-values (*p <* 0.05) are marked in bold print, and statistical trends are printed in italics. One asterisk is used to highlight correlations of trending significance. Two asterisks are used to highlight correlations with a significance level of *p <* 0.05.

### 3.4 Classification modeling

To assess the performance and diagnostic accuracy of clinical, cardiovascular, and ultrasound parameters and to identify interesting potential combinations of clinical, cardiovascular, and imaging parameters (ROC-models) with high diagnostic accuracy and discriminatory ability between CRP+ and CRP− groups, we next carried out random forest classification modeling with a greedy feature selection procedure under 5-fold cross-validation. We then performed permutation testing with 1,000 permutations to calculate the *p*-value of the ROC-AUC scores. Results are summarized in Table 4. As single ROC-models, SSc duration, total cholesterol, and HDL cholesterol demonstrated each a cross-validated AUC of 0.73, 0.75, and 0.76, respectively (0.005 ≤ *p* ≤ 0.011) and therefore showed good discriminatory ability to differentiate CRP−positivity in SSc patients. LDL cholesterol and ASCVD group assignment alone showed an AUC of 0.67 and 0.65, respectively, both approaching statistical significance (0.057 ≤ *p* ≤ 0.068). When looking at possible combinations of clinical and cardiovascular parameters, the greedy feature search procedure using the random forest classification model identified several combined ROC models with very high and significant cross-validated AUCs. These included a combined ROC model consisting of the four lipids: total cholesterol, HDL cholesterol, LDL cholesterol, and total triglycerides resulting in an AUC of 0.83 (model 1, *p <* 0.001). Moreover, combining SSc duration and ASCVD group into a combined ROC model yielded a significant AUC of 0.86 (model 2, *p <* 0.001). When the presence of type 2 diabetes mellitus was added, the significant cross-validated AUC of model 2 amounted to 0.85 (model 3, *p <* 0.001). All three combined ROC models showed a similarly high AUC. Combined ROC models using ultrasound parameters or the SCORE 2019 risk score did not reach comparably high AUC values (data not shown).

**Table 4:** Area under the ROC curve (AUC) values for different ROC models including combined ROC models.

Single ROC models	5-fold cross-validation AUC ± SD	Permutation test *p*-value
Age [years]	0.46 ± 0.07	0.625
Weight [kg]	0.46 ± 0.10	0.660
BMI [kg/m^2^]	0.64 ± 0.20	0.119
Sex, female n [%]	0.60 ± 0.15	0.101
SSc duration [months]	0.73 ± 0.12	**0.011**
Age at SSc diagnosis [years]	0.37 ± 0.12	0.915
Overall SSc organ involvement ł *n* [%]	0.58 ± 0.15	0.265
Diabetes mellitus type 2 (T2DM) *n*	0.58 ± 0.13	0.072
Total cholesterol [mg/dl]	0.75 ± 0.11	**0.010**
HDL cholesterol [mg/dl]	0.76 ± 0.15	**0.005**
LDL cholesterol [mg/dl]	0.67 ± 0.16	*0.057*
Triglycerides [mg/dl]	0.52 ± 0.12	0.412
Individual FRS 10-year risk of HCHD [%]	0.45 ± 0.25	0.647
Average FRS 10-year risk of HCHD [%]	0.60 ± 0.06	0.180
ASCVD 10-year risk [%]	0.47 ± 0.10	0.612
ASCVD risk group	0.65 ± 0.21	*0.068*
SCORE 2019 10-year risk of fatal CVD [%]	0.49 ± 0.13	0.550
CIMT right [mm]	0.46 ± 0.08	0.675
number of plaques right n	0.54 ± 0.06	0.352
maximal plaque diameter right [mm]	0.49 ± 0.18	0.380
CIMT left [mm]	0.37 ± 0.10	0.899
number of plaques left *n*	0.54 ± 0.23	0.391
maximal plaque diameter left [mm]	0.46 ± 0.12	0.667
Total number of plaques left and right n	0.54 ± 0.06	0.353
Mean CIMT left and right carotids art. [mm]	0.45 ± 0.14	0.621
Combined ROC models		
Model 1: Total cholesterol +HDL+ LDL + total triglycerides	0.83 ± 0.15	**<0.001**
Model 2: SSc duration +ASCVD group	0.86 ± 0.09	**<0.001**
Model 3: SSc duration + T2DM + ASCVD group	0.85 ± 0.05	**<0.001**

ASCVD, Arteriosclerotic cardiovascular disease; CVD, cardiovascular disease; CIMT, carotid intima–media thickness measured via ultrasound; FRS, Framingham risk score; HCDH, Hard coronary heart disease defined as either myocardial infarction or coronary death; HDL cholesterol, high-density lipoprotein cholesterol; LDL cholesterol, Low-density lipoprotein cholesterol; SSc, systemic sclerosis; T2DM, diabetes mellitus type 2; SCORE, Systematic cardiovascular risk evaluation. ASCVD risk groups encompassing the following four categories: low ASCVD risk group (containing SSc patients with < 5% calculated 10-year ASCVD risk score), borderline ASCVD risk group (containing SSc patients with a 5–7.4% calculated 10-year ASCVD risk score), intermediate ASCVD risk group (containing SSc patients with a 7.5–19.9% calculated 10-year ASCVD risk score), and high ASCVD risk group (containing SSc patients with a ≥ 20% calculated 10-year ASCVD risk score). Significant *p*-values (*p <* 0.05) are marked in bold print, and statistical trends are printed in italics.

## 4 Discussion

In this cross-sectional observational study, we aimed to explore and assess which general, SSc-specific, and cardiovascular parameters are associated with persistent CRP elevation and thus with chronic low-grade inflammation in SSc. In addition, we also investigated whether persistent CRP elevation in SSc patients is associated with a higher cardiovascular risk as determined using the Framingham risk and ASCVD risk scores and an increased atherosclerotic burden as determined via carotid ultrasound. Finally, we applied random forest classification modeling with cross-validation and permutation testing in order to identify combinations of cardiovascular and SSc-specific parameters that could best differentiate the two groups.

One of the main findings of our study is that a persistent CRP−positivity (of >5 mg/L for at least 1.5 consecutive years), and thus, chronic low-grade inflammation in SSc patients is associated with a significantly higher estimated 10-year risk for ASCVD risk than SSc patients without CRP elevations. The ASCVD risk estimation is based on sex- and age-specific Pooled Cohort Equations validated in Caucasian asymptomatic individuals (33, 36) and is therefore a rather conservative estimate. In a recent population-based cohort study, Kurmann et al. (37) demonstrated that for SSc patients in general (no stratification by CRP status yet), the real-world 10-year-risk for suffering a cardiovascular event was 5 times higher than what was predicted by their respective ASCVD risk scores (37), concluding that the currently recommended ASCVD risk calculator heavily underestimates the ASCVD risk in SSc. The mean ASCVD risk scores reported in Kurmann’s study was approximately 8.9% for all SSc patients (37), a magnitude comparable to the ASCVD-risk score magnitude reported in our study for our CRP− group (see Table 1). Given that Kurmann et al. (37) only looked at all SSc patients (without stratifying by CRP−positivity) and that our reported ASCVD risk for the CRP+ SSc patient group ranged with a mean risk of 15% even significantly above Kurmann’s SSc patient cohort ASCVD risk, it seems very likely that the significant relationship between prolonged CRP−positivity and 10-year arteriosclerotic-cardiovascular risk observed in our study may even be underestimated. Along these lines, Kurmann et al. (37) also found the FRS score to strongly underestimate CV risk in SSc patients by approximately 4-fold (37). Although we did not detect any significant association between FRS scores and CRP−positivity, it seems possible that our FRS-risk estimations for the CRP+ SSc group may have been overly conservative representations of hard coronary heart disease risk and thus may have obscured a potentially significant association with CRP levels in this specific patient group. Larger prospective population-based SSc studies stratified by persistent CRP positivity are therefore needed to validate and elucidate our findings further.

The mechanisms underlying the higher ASCVD risk in CRP+ SSc patients remain unclear but may be attributable in part to their chronic low-grade inflammatory state (38–40) and their disrupted lipid profile, higher BMI, and a more frequent glucocorticoid usage. Chronic inflammation (41) and particularly chronic-low-grade inflammation (42) has widely been accepted as important drivers of atherosclerosis in both non-SSc and SSc settings, leading to activation of leucocytes, oxidative stress, and endothelial dysfunction, triggering a cascade of events culminating in the disruption of atherosclerotic plaques (43–46). Particularly, CRP has been shown in a multitude of studies in non-SSc populations to predict the risk of (cardio)vascular disease independent of all traditional risk factors (47–49) and has emerged as a validated measure of cardiovascular inflammation of similar rank and importance as blood pressure or cholesterol (39). Despite this, CRP has been surprisingly underutilized in the field of primary care and cardiology as a determinant for vascular risk (39). In addition, in the rheumatologic field, SSc studies stratifying by elevated CRP status have been very scarce to date and have been limited so far to the analyses by Muangchan et al. (3) and Jha et al. (7)using data from the Canadian Scleroderma Research Group (3, 7). Although both authors did not focus on arteriosclerotic cardiovascular disease as the main outcome, Jha et al. (7) clearly demonstrated a significantly increased risk of death and pulmonary arterial hypertension in the presence of abnormally elevated CRP levels for SSc patients. Similar to our study, Muangchan et al. (3) observed a significantly higher BMI and more frequent corticosteroid usage in SSc patients with sustained CRP levels above 8 mg/L (3). Overall, both reports reinforce our current and previous (5) notion that chronic low-grade inflammation seems to be an important risk factor in SSc patients and that SSc patients with sustained CRP elevations should be classified as a separate “inflammatory” SSc phenotype.

Other conditions such as dyslipidemia, diabetes, and obesity have also been attributed to trigger and sustain inflammatory responses and thus contribute to atherosclerosis and ASCVD risk (41, 42). Interestingly, our CRP+ SSc group displayed a disrupted atherogenic lipid profile compared to their CRP− SSc peers and to recommended reference values. This altered lipid profile was characterized by increased borderline high to high levels of triglycerides, significantly reduced and borderline-low HDL cholesterol levels, and significantly increased borderline high to high LDL cholesterol levels. Moreover, combining all 4 lipids (HDL, LDL, total cholesterol, and triglycerides) into a classification ROC model was highly discriminatory to CRP−positivity. Abnormal lipid profiles have been described in SSc patients versus controls before (20, 50) and have been partly attributed to a reduced HDL cholesterol efflux capacity in SSc macrophages and thus an impaired ability of SSc patients to clear off cholesterol from the body (51). However, this is the first study to identify a strongly disrupted lipid profile as one of the main differentiating features of the inflammatory SSc phenotype. What drives the alteration in lipid profile in the CPR+ SSc group remains unknown. However, more and more experimental and clinical evidence in non-SSc patients hints toward a close interplay and a series of pathways linking inflammatory molecules such as CRP with lipid metabolism (41, 52, 53) which can both be pharmacologically targeted. Several non-SSc studies have demonstrated that, for example, statins are highly effective not only in lowering lipids but also in reducing CRP serum levels independently of effects on lipids (54, 55). As our patients in the CRP+ SSc group exhibited both elevated CRP levels and an abnormal lipid profile, one might speculate that this specific patient group may particularly benefit from the dual anti-inflammatory and lipid-lowering effects of lipid-lowering therapy. However, additional clinical trials of lipid-lowering agents in SSc patients are needed to prove the potential benefit of statins for this specific patient group.

Apart from the findings above, we also observed moderate-to-strong correlations between prolonged CRP−positivity in SSc patients with several carotid ultrasound parameters, especially with the left or/and right CIMT and with the number of arteriosclerotic plaques at the right carotid, suggesting an increasing subclinical atherosclerotic burden in inflammatory SSc patients. Our results extend to the exploratory observations by Gamal et al. (21) who reported CRP−positivity as a potential risk factor for atherosclerosis in SSc patients compared to controls (21). However, Gamal’s study neither reported the magnitude of CRP levels nor stratified by prolonged CRP status as CRP measurements were taken only at one time point (21). In line with our study, Schiopu et al. (19) and Sedky et al. (20) also observed in their SSc cohorts a significant association between CRP levels and carotid plaques number (19) and CIMT levels, respectively (20). However, unlike our study, both studies did not stratify their cohorts by CRP status due to limitations in the study design including the lack of serial CRP measurements (19).

Our study has several strengths such as a relatively large and well-characterized SSc patient cohort. In addition, our study design and patient selection were based on serial CRP measurements in order to identify an “inflammatory subtype” with a prolonged and thus chronic inflammation. During patient selection, we were also able to track patients for incurring infections and were able to exclude patients with elevated CRP levels due to concomitant infections.

Our study has also several limitations. The first limitation of our study pertains to the cross-sectional study design which only allowed for ASCVD risk estimation but did not provide any information on observed prospective ASCVD outcomes such as myocardial infarction. Second, a higher proportion of corticosteroid users and type 2 diabetics among the CRP+ SSc group may also have contributed to the higher amount of inflammation and subsequently the higher ASCVD risk noted in the CRP+ SSc group. However, due to the limited sample size further subgroup analyses, adjustments for confounding factors, especially further stratification by immunosuppressive medication usage, were not possible. To minimize these drawbacks, we tried to be as transparent as possible by calculating correlations separately for the groups, by reporting extensively group characteristics and group comparisons on all variables of interest, and by cautiously interpreting our findings. Third, we were only able to assess the atherosclerotic burden at the carotid artery level via ultrasound and did not have the means to quantify the extent of atherosclerosis at other ASCVD predilection sites such as the coronary arteries (via calcium-scoring). Moreover, unlike Pagkopoulou et al. (56), we did not have nail fold video capillaroscopy at disposition at our study visit and therefore could not assess morphological markers of microcirculatory dysregulation. Therefore, our study might have underestimated the true extent of microvascular damage present in our SSc patients. However, we collected information on the presence and history of digital ulcerations, PAH, and Raynaud’s syndrome through visual inspection and physical examination during our study visits and by chart review. As those standard vascular parameters showed comparable proportions across both CRP groups, we are confident that the significant association between ASCVD risk and CRP positivity is not mainly driven by a higher vascular dysfunction in the CRP+ group.

## 5 Conclusion

While having an autoimmune disease such as SSc alone increases the risk of experiencing a CV event (37) and for coronary heart disease by 3-fold (57) compared to the general population, our study provides first hints that suffering from SSc and having additionally a chronic inflammation as seen in our CRP+ SSc group may enhance the estimated risk for arteriosclerotic cardiovascular disease beyond the risk of CRP− SSc patients. Prolonged CRP−positivity may act as an additional important modifiable risk-enhancing factor for atherosclerotic-cardiovascular disease in patients with systemic sclerosis and seems to be along with a disrupted lipid profile the hallmark of a distinct “inflammatory” subgroup of SSc patients who are more prone to suffer from musculoskeletal involvement as well as to a higher cardiovascular risk. SSc patients might therefore benefit from serial tracking of inflammatory markers such as CRP and from lipid markers as part of their standard surveillance and follow-up regimen. Due to the dual anti-inflammatory and lipid-lowering effect of statins, those SSc patients may even benefit from aggressive lipid-lowering and anti-inflammatory pharmacological therapies. However, large population-based studies and clinical trials in patients with SSc are needed to validate our findings in a prospective or interventional setting.

## Data Availability

The datasets presented in this article are not readily available because of ethical and privacy restrictions. Requests to access the datasets should be directed to SF, stephanie.finzel@uniklinik-freiburg.de.
