# Supplementary material for: Chronic low-grade inflammation in patients with systemic sclerosis is associated with increased risk for arteriosclerotic cardiovascular disease
Source: Front Med (Lausanne). 2024 Nov 5;11:1446268. doi: 10.3389/fmed.2024.1446268 (PMC11573558; doi:10.3389/fmed.2024.1446268)
Supplement: Supplementary file 1 [file Table_1.DOCX]

Supplementary Material

# Supplementary Data

**Supplementary Table 1 Legend:**

Patient characteristics of a study subset of n=49 patients with systemic sclerosis (SSc) stratified by ASCVD-risk status. Patients with 10y-ASCVD risk scores of < 5% were assigned to the SSc-Low risk group, while SSc patients with an ASCVD risk score of (≥7.5%) or > 20% were assigned to the combined intermediate/high risk-SSc group (IMH-risk) Shown are unadjusted means with standard deviation (SD). Intergroup differences were calculated using Mann-Whitney-U test or with independent t-tests or Chi-Square tests or Fisher exact tests as appropriate. Significant p-values (p<0.05) are marked in bold print, statistical trends are printed in italics.

**Supplementary Table 2 Legend:**

Results of binary logistic regression analyses carried out in all n= 65 SSc patients to assess associations between arteriosclerotic carotid ultrasound parameters and CRP-Positivity status. CRP-status was considered positive or negative respectively, if at least 75% of the highly sensitive CRP values were positive (> 5 mg/l) or negative (≤ 5 mg/l) in at least three half-yearly visits within the past two years prior to study start. An Odds ratio (OR) greater than 1 indicates that a higher value in the respective candidate predictor variable (or response ‘yes’ for categorical candidate predictor variables) is associated with a higher probability of belonging to group CRP+. Significant p-values (p<0.05) would be marked in bold print.

| **Supplementary Table 1.** | **Means ± SD** | |  |
| --- | --- | --- | --- |
|  | **Low-risk group**  **(n=23)** | **IMH-risk group**  **(n= 26)** | **p-value** |
| ***Demographics*** |  |  |  |
| Age [years] | 50.0 ± 7.2 | 66.9 ± 9.3 | **<0.001** |
| BMI [kg/m^2^] | 25.3 ± 5.3 | 27.6 ± 4.3 | **0.068** |
| Height (m) | 1.7 ± 0.1 | 1.7 ± 0.10 | 0.669 |
| Weight [kg] | 69.7 ± 15.4 | 77.4 ± 16.6 | **0.104** |
| Gender, female n [%] | 21 [91.3] | 18 [69.2] | *0.056* |
| ***SSc characteristics and medication usage*** |  |  |  |
| Time since SSc diagnosis [months] | 102.4 ± 57.8 | 137.9 ± 104.4 | 0.383 |
| Age of SSc diagnosis [years] | 41.5 ±7.3 | 55.4 ± 9.4 | **<0.001** |
| Overall organ involvement ł n [%] | 10 [43.5] | 20 ± [76.9] | **0.016** |
| Number of organs involved ł n [%] | 0.91 ± 1.1 | 1.35 ± 1.23 | 0.145 |
| Heart involvement n [%] | 1 [4.3] | 2 [7.7] | 0.626 |
| ***Serum laboratory measures*** |  |  |  |
| CRP levels [mg/l] | 3.9 ± 1.5 | 7.1 ± 6.0 | **0.024** |
| eGFR (estimated glomerular filtration  rate) [ml/min/1.73m^2^] | 87.0  [79.2 – 92.5] | 66.5  [57.3 – 72.7] | **<0.001** |
| ***Cardiovascular risk factors*** |  |  |  |
| Arterial hypertension n [%] | 8 [34.8] | 18 [69.2] | **0.016** |
| History of smoking n [%] | 9 [39.1] | 17 [65.4] | *0.066* |
| Packyears [years] | 4.5 ± 8.4 | 13.9 ± 18.2 | **0.031** |
| Diabetes mellitus type 2 n [%] | 0 [0.0] | 4 [15.4] | ***0.050*** |
| Chronic kidney disease n [%] | 2 [8.7] | 7 [26.9] | 0.100 |
| Positive family history for cardio-  vascular event < age of 65 years n [%] | 3 [13.0] | 6 [23.1] | 0.365 |
| Individual FRS 10-year risk of HCHD~ [%] | 1.79 ± 2.1 | 11.5 ± 7.0 | **<0.001** |
| Average FRS 10-year risk of HCHD ~ [%] | 4.3 ± 3.3 | 10.9 ± 5.2 | **<0.001** |
| 10-year ASCVD risk [%] | 1.7 ± 1.1 | 20.8 ± 9.2 | **<0.001** |
| ***Arteriosclerotic assessment by carotid US*** |  |  |  |
| **Right side carotid arteries** | (n= 23) | (n=25) |  |
| CIMT right [mm] | 0.56 ± 0.13 | 0.68 ± 0.23 | **0.030** |
| proportion of patients with right plaques [%] | 6 [26.1] | 15 [60.0] | **0.018** |
| number of plaques right | 0.26 ± 0.20 | 0.72 ± 0.68 | **0.012** |
| maximal plaque diameter [mm] | 0.49 ± 0.96 | 1.4 ± 1.3 | **0.007** |
| **Left side carotid arteries** | (n= 19) | (n=44) |  |
| CIMT left (mm) | 0.52 ± 0.16 | 0.78 ± 0.32 | **0.001** |
| proportion of patients with left plaques [%] | 6 [26.1] | 15 [60.0] | **0.018** |
| number of plaques left | 0.17± 0.39 | 0.75 ± 0.53 | **<0.001** |
| maximal plaque diameter [mm] | 0.33 ± 0.74 | 1.73 ± 1.49 | **<0.001** |
| **Total number of plaques left and right (N)** | 1.25 ± 0.46 | 1.80 ± 0.70 | **0.049** |
| Ł organ involvement was considered present if any of the following organs (esophagus, lung, heart, GI-system, or kidney) were affected by SSc.  ASCVD = Arteriosclerotic cardiovascular disease. CIMT = carotid intima-media thickness measured via ultrasound.  FRS = Framingham Risk Score. ~HCDH = hard coronary heart disease defined as either myocardial infarction or coronary death. HDL cholesterol = High-density lipoprotein cholesterol.  LDL cholesterol = low density lipoprotein cholesterol.  SSc= Systemic sclerosis. T2DM = diabetes mellitus type 2. | | | |

| **Supplementary Table 2:** | **OR** | **95% CI of OR** | **p-value** |
| --- | --- | --- | --- |
| ***Arteriosclerotic carotid ultrasound parameters*** | | | |
| CIMT right [mm] | 3.54 | [0.26, 48.49] | 0.344 |
| proportion of patients with right carotid  plaques n [%] | 2.17 | [0.73; 6.37] | 0.160 |
| number of plaques right n | 1.71 | [0.75, 3.91] | 0.200 |
| maximal plaque diameter right [mm] | 1.41 | [0.91, 2.18] | 0.119 |
| CIMT left [mm] | 2.02 | [0.28, 14.51] | 0.484 |
| proportion of patients with left carotid  plaques [%] | 2.18 | [0.73, 6.52] | 0.162 |
| number of plaques left n | 1.59 | [0.61, 4.10] | 0.341 |
| maximal plaque diameter left [mm] | 1.11 | [0.77, 1.59] | 0.583 |
| Total number of plaques left and right n | 1.66 | [0.64, 4.32] | 0.302 |
| Mean CIMT left and right carotids art. [mm] | 3.53 | [0.29; 43.4] | 0.325 |
| CIMT= carotid intima-media thickness measured via ultrasound. | | | |

**
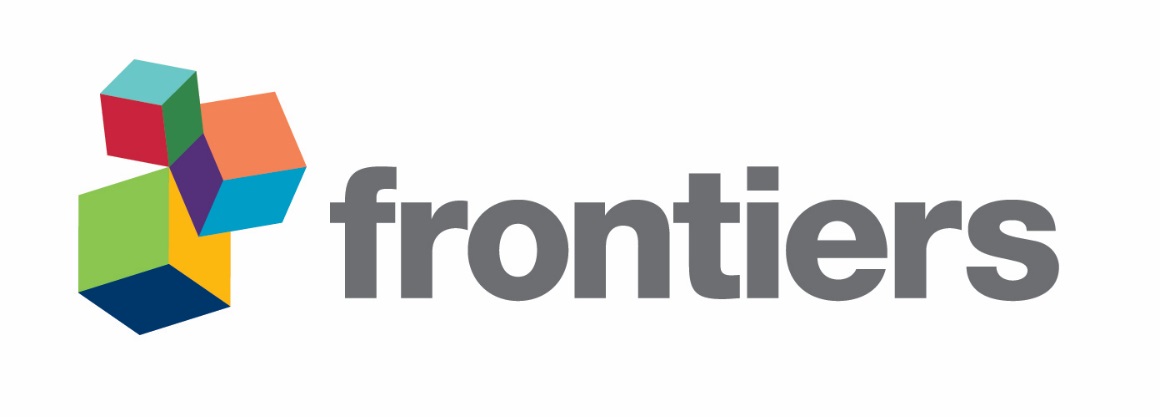
**
